# Antibody and Nanobody Radiolabeling with Copper-64: Solid vs. Liquid Target Approach

**DOI:** 10.3390/molecules28124670

**Published:** 2023-06-09

**Authors:** Ivanna Hrynchak, Diana Cocioabă, Alexandra I. Fonseca, Radu Leonte, Sérgio J. C. do Carmo, Roxana Cornoiu, Amílcar Falcão, Dana Niculae, Antero J. Abrunhosa

**Affiliations:** 1 Institute for Nuclear Sciences Applied to Health (ICNAS Pharma), Polo das Ciências da Saúde, University of Coimbra, 3000-548 Coimbra, Portugal; ivanna.ua@icnas.uc.pt (I.H.); alexandrafonseca@icnas.uc.pt (A.I.F.); sergiocarmo@uc.pt (S.J.C.d.C.); amilcar.falcao@uc.pt (A.F.); 2 Horia Hulubei National Institute for Physics and Nuclear Engineering (IFIN-HH), Radiopharmaceutical Research Centre, 077125 Măgurele, Romania; diana.cocioaba@nipne.ro (D.C.); radu.leonte@nipne.ro (R.L.); roxana.cornoiu@nipne.ro (R.C.); dana.niculae@nipne.ro (D.N.); 3 Faculty of Physics, Doctoral School of Physics, University of Bucharest, 077125 Bucharest, Romania; 4 Coimbra Institute for Biomedical Imaging and Translational Research (CIBIT), University of Coimbra, 3000-548 Coimbra, Portugal; 5 Institute for Nuclear Sciences Applied to Health (ICNAS), University of Coimbra, 3000-548 Coimbra, Portugal; 6 Faculty of Chemical Engineering and Biotechnologies, Doctoral School of Applied Chemistry and Materials Science, University Politehnica of Bucharest, 011061 Bucharest, Romania; 7 Faculty of Pharmacy, University of Coimbra, 3000-548 Coimbra, Portugal

**Keywords:** copper-64, liquid target, solid target, radiolabeling, antibodies, nanobodies

## Abstract

Antibody and nanobody-based copper-64 radiopharmaceuticals are increasingly being proposed as theranostic tools in multiple human diseases. While the production of copper-64 using solid targets has been established for many years, its use is limited due to the complexity of solid target systems, which are available in only a few cyclotrons worldwide. In contrast, liquid targets, available in virtually in all cyclotrons, constitute a practical and reliable alternative. In this study, we discuss the production, purification, and radiolabeling of antibodies and nanobodies using copper-64 obtained from both solid and liquid targets. Copper-64 production from solid targets was performed on a TR-19 cyclotron with an energy of 11.7 MeV, while liquid target production was obtained by bombarding a nickel-64 solution using an IBA Cyclone Kiube cyclotron with 16.9 MeV on target. Copper-64 was purified from both solid and liquid targets and used to radiolabel NODAGA-Nb, NOTA-Nb, and DOTA-Trastuzumab conjugates. Stability studies were conducted on all radioimmunoconjugates in mouse serum, PBS, and DTPA. Irradiation of the solid target yielded 13.5 ± 0.5 GBq with a beam current of 25 ± 1.2 μA and an irradiation time of 6 h. On the other hand, irradiation of the liquid target resulted in 2.8 ± 1.3 GBq at the end of bombardment (EOB) with a beam current of 54.5 ± 7.8 μA and an irradiation time of 4.1 ± 1.3 h. Successful radiolabeling of NODAGA-Nb, NOTA-Nb, and DOTA-Trastuzumab with copper-64 from both solid and liquid targets was achieved. Specific activities (SA) obtained with the solid target were 0.11, 0.19, and 0.33 MBq/μg for NODAGA-Nb, NOTA-Nb, and DOTA-trastuzumab, respectively. For the liquid target, the corresponding SA values were 0.15, 0.12, and 0.30 MBq/μg. Furthermore, all three radiopharmaceuticals demonstrated stability under the testing conditions. While solid targets have the potential to produce significantly higher activity in a single run, the liquid process offers advantages such as speed, ease of automation, and the feasibility of back-to-back production using a medical cyclotron. In this study, successful radiolabeling of antibodies and nanobodies was achieved using both solid and liquid targets approaches. The radiolabeled compounds exhibited high radiochemical purity and specific activity, rendering them suitable for subsequent in vivo pre-clinical imaging studies.

## 1. Introduction

Copper-64 is a radioisotope that possesses favorable physical characteristics for positron emission tomography (PET). It has a T_1/2_ = 12.7 h, β^+^_avg_ = 278 KeV, β^+^_max_ = 652.9 KeV, and β^+^_abundance_ = 17.87% [1]. Additionally, copper-64 also emits β^−^ particles (38.48%) and generates Auger electrons through electron capture (43.53%), enabling it to be used for radiotherapy [2,3].

Among the various nuclear reactions studied, the ^64^Ni(*p*,*n*)^64^Cu reaction is the preferred route [4,5] due to its ability to achieve a high production yield of copper-64 using low-energy protons, even though the target material is expensive. Comparably to other radioisotopes, copper-64 should be administered in the form of a thermodynamically stable and kinetically inert complex to prevent nonspecific binding [6,7]. The versatile coordination chemistry of copper-64 enables its reaction with a wide range of chelators including NOTA, DOTA, or NODAGA. These chelators can be linked to peptides [8,9], nanoparticles [10,11], and antibodies [12,13]. As a result, production routes for copper-64 have been widely explored using both solid [4,14] and liquid targets [15,16]. However, the routine production of radiometals using solid targets at clinical PET facilities poses challenges, particularly in terms of automating the transfer, purification, and labeling processes [17]. In 2017, Alves et al. developed a successful production process of copper-64 using liquid targets. The approach yielded a purified solution suitable for labeling radiopharmaceuticals intended for human use. The final activities obtained were enough to enable the distribution of copper-64 to other PET centers [15].

Recently, human studies have been focusing on the use of ^64^Cu-labeled bioconjugates which include antibodies and antibody fragments such as nanobodies for patient-specific treatment improvement. Monoclonal antibodies are of particular interest due to their exceptional target affinity and specificity [18]. Several ^64^Cu-labeled antibodies have been developed for PET imaging applications. These include trastuzumab, used for imaging breast cancers expressing human epidermal growth factor receptor 2 (EGFR-2 or HER2), cetuximab which targets tumors expressing EGFR receptor, TRC105-Fab for CD105 targeting, and etaracizumab, an antibody used against human 𝛼_v_𝛽_3_ integrin [19].

The widely studied trastuzumab, commonly chelated with DOTA, is a validated targeting vector. It is also a good model for comparing the radiolabeling properties of our novel nanobody (ICNAS-01) using NOGADA and NOTA as chelating agents. Herein we describe the feasibility of using both approaches, solid and liquid targets, to produce copper-64 radionuclide precursor for radiolabeling immunoconjugates. Additionally, production, copper-64 purification yield, and radiolabeling methods using copper-64 produced from both solid and liquid targets are discussed.

## 2. Results and Discussion

### 2.1. Copper-64 Production from Solid Targets

Targets were successfully prepared by electroplating approximately 50 mg of nickel-64 in an aqueous solution at 2.7–2.9 V, with a starting current intensity of 37 mA, for 20–24 h. The end of the electrodeposition process was indicated by the complete change of color of the electroplating solution from dark blue to colorless. After cleaning and drying, we obtained a circular-shaped target on the shuttle, containing 48 ± 1 mg nickel-64, ready for bombardment [20].

Copper-64 produced using a solid target on the TR-19 cyclotron was obtained with an integrated current of 150 μAh at 11.7 MeV on target/14.2 MeV extracted, resulting in 5.7–6.2 GBq at the time of radiolabeling, 15 h from the end of bombardment (EOB), (n = 2, Table 1). The activity corresponds to 90 ± 3.4 MBq/μAh at EOB. For solid target irradiation, the produced amount is highly dependent on the precision of the beam focusing on the target area, the beam shape, and position being checked on a test paper before irradiation and adjusted if necessary.

#### 2.1.1. Copper-64 Purification

After 6 h of irradiation, the dissolution and purification steps were conducted on EDS and TADDEO-PRF automatic modules, respectively. The irradiated nickel-64 target was dissolved directly in the platinum well of the shuttle with 6 M HCl and the resulting [^64^Cu]CuCl_2_ solution was loaded onto an anion exchange column AG1-X8 to separate copper from nickel, cobalt, and other metallic impurities. The ^64^Ni fraction was collected with 6 M HCl and the metallic impurities were washed with 4 M HCl. Copper-64 was eluted from the column with 0.5 M HCl in three different fractions aiming to concentrate the radioactivity; the second fraction, containing most of the activity, was further used for radiolabeling. Subsequently, [^64^Cu]CuCl_2_ solution was evaporated to dryness and redissolved in water. The dissolution and separation processes take around 50 min, with >96% yield. The solid target recovery process described in the references [21,22], which yields above 90%, was followed.

#### 2.1.2. Quality Control

**Figure 1:**
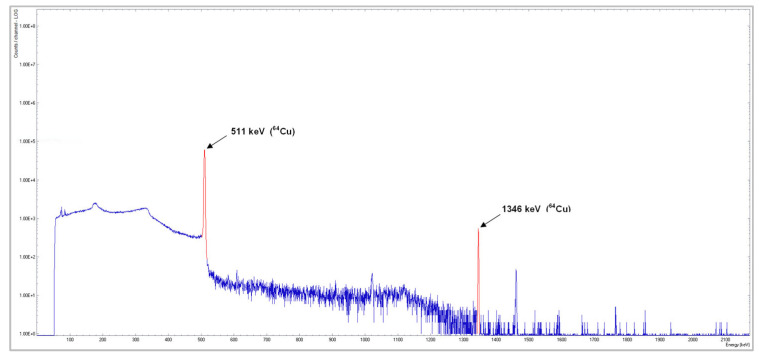
Gamma-ray spectrum of the [^64^Cu]CuCl_2_ solution resulting from solid target.

The quality of [^64^Cu]CuCl_2_ was assessed at the end of the purification step. The radionuclidic purity and identification were assessed by gamma-ray spectrometry using a gamma-spectrometer equipped with an HPGe detector (Baltic Scientific Instruments, Riga, Latvia). The gamma spectrum (Figure 1) showed that the copper-64 radionuclidic purity was higher than 99.99%.

The half-life determination was performed using a dose calibrator and associated software (Veenstra-Comecer, The Netherlands), indicating a value of 12.43 ± 0.2 h in accordance with the reference value (12.7 ± 0.6 h), which confirms the identity of copper-64. The radiochemical purity assessed by iTLC was 100% (Figure S1) using sodium citrate 0.1 M as the mobile phase and silica gel 60 on aluminum sheets as the stationary phase (Merck KGaA, Darmstadt, Germany). The presence of radioactive colloidal forms was not observed.

### 2.2. ^64^Cu Production from Liquid Targets

The irradiation of liquid targets containing enriched nickel (>95.0% isotopic enrichment) to produce copper-64 of high radionuclidic purity is performed on a routine basis at ICNAS (University of Coimbra, Portugal). A 3–5 h long irradiation using an IBA Cyclone Kiube Variable energy cyclotron with 18 MeV extracted beam energy, corresponding to 16.9 MeV on target, can produce up to 5.1 GBq of copper-64 (Table 1).

**Figure 2:**
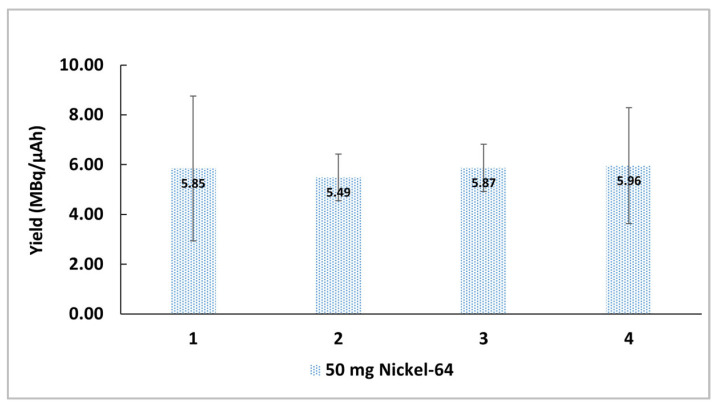
Production yield (MBq/µAh) obtained from four consecutive production runs using recycled nickel-64 (n = 4).

As the production of copper-64 requires a large initial investment in enriched nickel-64, the target material is recovered and reused. The recycling process from liquid targets is simple and reliable, making the routine production of copper-64 a more cost-effective process. As it can be concluded by the analysis of the production yield (MBq/µAh) of copper-64 shown in Figure 2, the reused nickel-64 allows for reproducible copper-64 productions without affecting the radionuclidic purity or decrease in the production yield. In short, the production of copper-64 using recycled nickel-64 liquid targets overcomes the practical difficulties associated with the recycling process of solid targets [16] and makes the production of copper-64 more cost effective.

#### 2.2.1. ^64^Cu Purification

**Figure 3:**
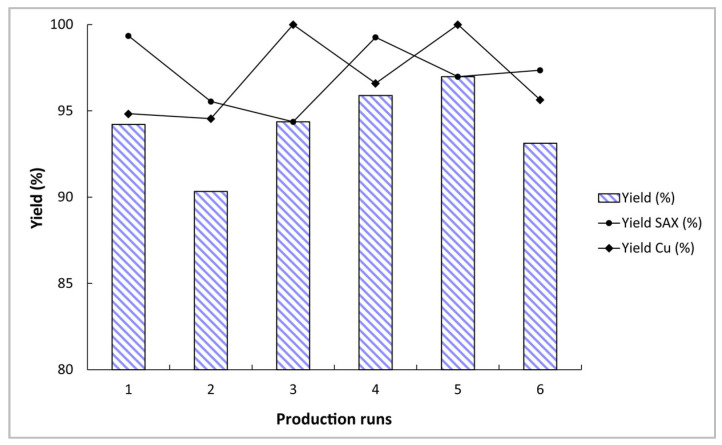
Six consecutive purification runs. The black lines detail the recovery yield of each step, whereas the bars represent the overall process recovery yield.

The purification method used to separate copper-64 produced from nickel target material is similar to the purification process used for solid targets, but without the need for dissolution [16,23]. The two steps method was conducted using an IBA Synthera^®^ Extension module. First, a selective extraction chromatographic CU resin was used to separate copper-64 from nickel-64 and then the copper-64 was directly transferred onto a strong anion exchange (SAX) resin to eliminate trace quantities of metal contaminants and to reduce the HCl concentration on the final solution. The purified copper-64 was eluted with water to achieve the final [^64^Cu]CuCl_2_ ready to be used in a labeling process. This purification process was completed in less than 40 min from the EOB with copper recovery yield of 94.15 ± 2.31% (decay-corrected at the EOB). Figure 3 exemplifies purification yields of six consecutive purification runs. By the analysis of these results, it is possible to conclude that the purification process is highly reproducible, with overall yields consistently above 90%.

The metal impurities data in the final solution [^64^Cu]CuCl_2_ were previously published by our group [16]. Several metal contaminants such as Al, Fe, Ni, Cu, Zn, and Pb were analyzed using inductively coupled plasma mass spectrometry (ICP-MS). We found a very low presence of these metal contaminants (part per billion level). In this paper, we suggest that the aluminum originates from the glass storage container and the iron derives from the reagents used in the process.

#### 2.2.2. Quality Control

**Figure 4:**
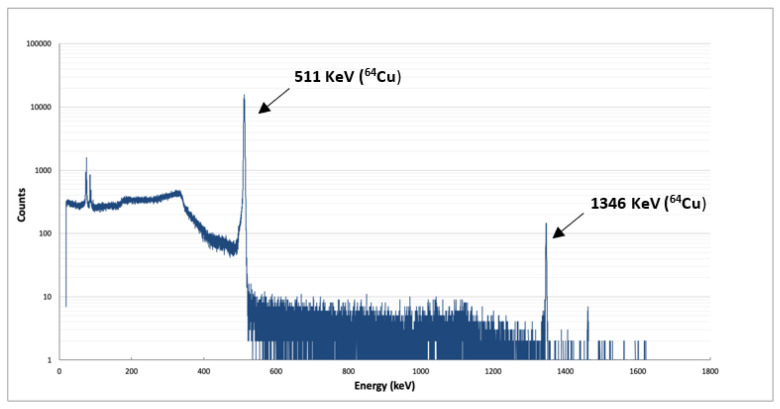
Gamma-ray spectrum of the [^64^Cu]CuCl_2_ solution resulting from liquid target.

The purity of the copper-64 produced from a cyclotron using the liquid target approach was investigated by determining the half-life of copper-64, as well as gamma-ray spectrometry. Similar to the ^64^Cu-solution obtained from solid target, the half-life was measured using a dose calibrator and the result was 12.37 ± 0.1 h, also in accordance with the reference value of 12.7 ± 0.6 h. The gamma spectrum (Figure 4) showed that the copper-64 radionuclidic purity was over 99% at end of purification. Additionally, the radiochemical purity assessed by iTLC was 100% (Rf = 1) using sodium citrate 0.1 M as mobile phase. The presence of a colloidal form was not observed (Rf = 0) (Figure S1).

### 2.3. Radiolabeling

A range of volumes of purified [^64^Cu]CuCl_2_ solution (~38 or 123 μL) obtained via the solid target route was incubated with varying quantities of conjugated DOTA-Trastuzumab, NODAGA-Nanobody, and NOTA-Nanobody at room temperature, at pH = 6 within 30 min. Radiolabeling efficiency was evaluated by iTLC and the specific activity (SA) was estimated based on the radiolabeling efficiency divided by the initial amount of the immunoconjugates. In the first experiment using 38 μL (94 MBq) of [^64^Cu]CuCl_2_ and 285 μg of DOTA-Trastuzumab, 882 μg of NODAGA-Nanobody, and 497 μg of NOTA-Nanobody, respectively, radiolabeling yields of 100% were obtained. Corresponding calculated specific activity (SA) was 0.33 MBq/μg, 0.11 MBq/μg, and 0.19 MBq/μg, respectively. Increasing the amount of [^64^Cu]CuCl_2_ to 123 μL (304 MBq) and also the amount of the immunoconjugates to 780 μg of DOTA-Trastuzumab, 1.48 mg of NODAGA-Nanobody, and 1.22 mg of NOTA-Nanobody, a decrease in the radiolabeling yield to 89.2% for the antibody was observed and, consequently, resulted in lower SA of 0.27 MBq/μg. The nanobodies followed the same high efficiency radiolabeling, of 100%, with an SA of 0.14 MBq/μg and 0.13 MBq/μg, respectively.

Aiming to compare the radiolabeling efficiency between copper-64 obtained from solid versus liquid targets, a similar amount of activity was used (~100 MBq). Volume-to-volume comparation was not conducted as a much higher amount of copper-64 activity is produced from solid target compared to liquid target. Therefore, a larger volume of [^64^Cu]CuCl_2_ solution obtained from the liquid target was used to match the activity of [^64^Cu]CuCl_2_ solution obtained from the solid target. Thus, volumes of [^64^Cu]CuCl_2_ solution in the range of 90–200 μL were used for radiolabeling of 281 μg DOTA-Trastuzumab, 881 μg NODAGA-Nanobody, and 630 μg NOTA-Nanobody, respectively. The radiochemical yields of the reactions were 97.4%, 93.57%, and 82.76%, assessed by iTLC, with SA of 0.29 MBq/μg, 0.15 MBq/μg, and 0.12 MBq/μg, respectively (Table 2 and Table S1). After purification on PD-10 column, the radiochemical purity in all three cases was 100%, assessed by iTLC. The radiochemical purity assessed by iTLC was consistent with radiolabeling results determined by size-exclusion HPLC (Figure S2). These similar results suggest that high-quality [^64^Cu]CuCl_2_, suitable for labeling monoclonal antibodies for PET imaging, can be obtained via the liquid target route. Additionally, both radiolabeling conditions resulted in SAs with values suitable for μPET imaging [24].

We tested two different molecular entities, an antibody and a nanobody, each functionalized with three different chelating agents. Despite several authors arguing that DOTA is an inconvenient chelator for antibodies due to its high temperature requirements [25,26,27,28], we have proved that it is a suitable chelator for copper. At room temperature, we obtained high radiochemical yields after 30 min using both solid and liquid target routes. Regarding nanobody radiolabeling, NODAGA was revealed to be the best chelating agent for copper-64 in our conditions.

Small differences can be noted in the radiochemical yields when comparing solid and liquid targets, which can be attributed to the higher concentration of copper-64 obtained from solid targets as compared to the liquid targets. Higher activity productions from the solid targets result in smaller volumes of [^64^Cu]CuCl_2_ solution being required, and consequently, higher concentrations in the radiolabeling reactions.

### 2.4. Stability Studies

**Figure 5:**
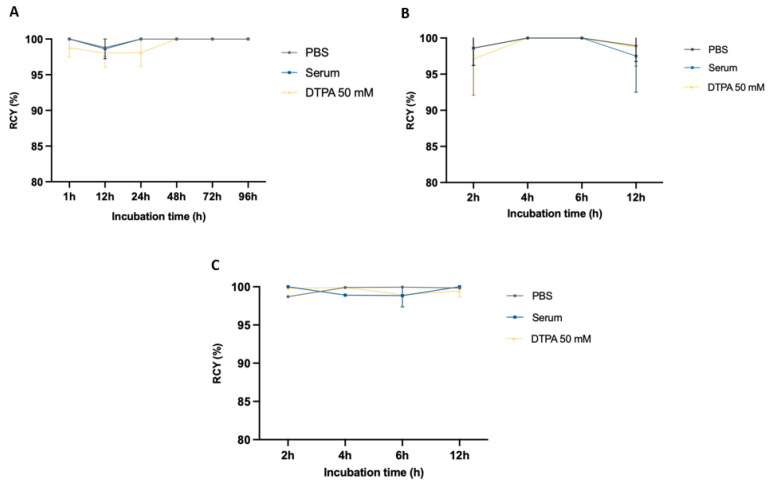
Stability of [^64^Cu]Cu-DOTA-Trastuzumab (**A**), [^64^Cu]Cu-NODAGA-Nanobody (**B**), and [^64^Cu]Cu-NOTA-Nanobody (**C**) in PBS, mice serum, and DTPA 50 mM, respectively. Radiochemical purity results were obtained by iTLC.

The stability of [^64^Cu]Cu-DOTA-Trastuzumab was evaluated in PBS, DTPA 50 mM, and mouse serum up to 7 days, while for nanobodies, [^64^Cu]Cu-NODAGA-Nanobody and [^64^Cu]Cu-NOTA-Nanobody, the stability was evaluated up to 12 h due to their shorter plasma half-life. Figure 5 shows that all three radiopharmaceuticals were stable under the testing conditions. The radiochemical purity, exceeding 95% during testing intervals, suggests that these compounds exhibit high stability for up to 196 h (full antibody) or 12 h (nanobody) of incubation in all mediums (PBS, DTPA 50 mM, and mouse serum).

## 3. Materials and Methods

### 3.1. Reagents and Instruments

All chemicals and solvents used for the synthesis and purification of [^64^Cu]CuCl_2_ and ^64^Cu-immunoconjugates were of metal trace grade, and HPLC solvents were HPLC grade. Enriched isotope nickel-64 (^64^Ni) was purchased from Fluidomica (Cantanhede, Portugal) and ISOFLEX (San Francisco, USA) in the form of metallic powder with >95.0% and >99.5% enrichment, respectively. Water (TraceSELECTTM, 99.9999999% metals basis) was obtained from Honeywell (Seelze, Germany). Hydrochloric acid (30% min, TraceSELECTTM, 99.9999999% metals basis) and nitric acid (69%, TraceSELECTTM, 99.9999999% metals basis) were obtained from Honeywell (Seelze, Germany). Bi-distilled water was purchased from B-Braun (Melsungen, Germany). CU Resin and anion exchange resin (SAX) were purchased from TrisKem International (Bruz, France) and AG1-X8 resin from Bio-Rad Laboratories (Hercules, CA, USA). The p-isothiocyanatobenzyl-1,4,7,10-tetraazacyclododecane tetraacetic acid (p-SCN-Bn-DOTA) was purchased from Macrocyclics and Trastuzumab (Herceptin) was purchased from Evidentic GmbH (Berlin, Germany). Additionally, 2,2′-(7-(2-((2-(2,5-dioxo-2,5-dihydro-1H-pyrrol-1-yl)ethyl)amino)-2-oxoethyl)-1,4,7-triazonane-1,4-diyl)diacetic acid (maleimide-NOTA) and 2,2′-(7-(1-carboxy-4-((2-(2,5-dioxo-2,5-dihydro-1H-pyrrol-1-yl)ethyl)amino)-4-oxobutyl)-1,4,7-triazonane-1,4-diyl)diacetic acid (maleimide-NODA-GA) were obtained from CheMatech (Dijon, France). The nanobody (ICNAS-01) was manufactured by Technophage (Lisbon, Portugal). Dimethyl sulfoxide (DMSO) used for chelator stock solutions and amicon ulta centrifugal filters (3 K and 100 K) were purchased from Sigma-Aldrich (Algés, Portugal). DL-Dithiothreitol (DTT) was obtained from GoldBio (St. Louis, MO, USA). All other chemicals were analytical grade and used without further purification.

The bicinchoninic acid assay (BCA) was performed on a 96-well plate and read in ELISA equipment at 562 nm. The radiolabeled immunoconjugates were monitored using silica-impregnated instant thin-layer chromatography paper (iTLC-SG) and analyzed on a Ray Test miniGita detector and Mini Ginastar software (Straubenhardt, Germany). The mobile phase was sodium citrate 0.1 M. After conjugation and radiolabeling, molecular size exclusion columns PD-10 (GE HealthCare, New York, NY, USA) were used for purification. High-performance liquid chromatography (HPLC) analyses of purified radioimmunoconjugates were carried out using a size-exclusion chromatography (SEC) column (SuperdexTM 200 10/300 GL, GE Healthcare, New York, USA) and conducted on an Agilent 1260 Infinity System equipped with an Agilent 1200 DAD UV detector (UV detection at 280 nm) (Santa Clara, CA, USA) equipped with Raytest radiation detector (Raytest GmbH, Straubenhardt, Germany). The HPLC method applied was isocratic constituted by of 0.1 M sodium phosphate monobasic, 0.1 M sodium phosphate dibasic, 0.1 M sodium azide, and 0.15 M sodium chloride (pH 6.2–7.0). Radionuclidic purity of the ^64^Cu was determined by gamma-spectroscopy using a High Purity Germanium detector (HPGe) from Ortec (ORTEC Inc., Oak Ridge, TN, USA), calibrated with europium-154 (^154^Eu) and barium-133 (^133^Ba) radioactive point-like sources. The GammaVision software (for Windows Model A66-B32 Version 6.07), also from ORTEC, was used to determine photopeak areas.

### 3.2. Production and Purification from Solid Target

#### 3.2.1. Target Preparation/Irradiation

Before irradiation, the target was obtained by electrodeposition of metallic nickel-64 (99.53%) onto platinum support based on the work of Leonte et al., 2020 [20].

Irradiation of solid target for production of copper-64 through ^64^Ni(p,n)^64^Cu nuclear reaction was performed on TR-19 variable energy cyclotron (Advanced Cyclotron Systems Inc., Vancouver, BC, Canada) from Horia Hulubei National Institute for Physics and Nuclear Engineering (IFIN HH), Măgurele, Romania. The electroplated target was sent to the irradiation station dedicated to solid targets and irradiated with 11.7 ± 0.6 MeV protons (on target) for 6 h at 25 μA beam intensity (parameters set). The proton beam was extracted at 14.2 ± 0.3 MeV and degraded by passing through a pure aluminum foil (320 μm thickness) before reaching the target surface [14]. After irradiation, the shuttle was transferred remotely to the dissolution station of EDS module. The dissolution was conducted in two steps with 6 M HCl at 90 °C for approximately 30 min, followed by washing with cold 6 M HCl. The resulting solutions containing [^64^Cu]CuCl_2_ were then transferred to the purification module TADDEO PRF (Comecer, Castel Bolognese, Italy).

#### 3.2.2. Purification

The crude solution obtained at the dissolution step was purified by the ion exchange technique [20]. The highly acidic solutions were passed through an AG1-X8 anion column aiming to separate the copper-64 from the rest of the metallic impurities and to recover the unreacted nickel-64. Before the elution of copper-64 with 0.5 M HCl, the rest of the metallic impurities were removed by washing the column with 4 M HCl. Finally, copper-64 was evaporated and then re-dissolved in water as the solution for radiolabeling. The purification process was completed within a maximum of 50 min from the EOB.

### 3.3. Production and Purification from Liquid Target

#### 3.3.1. Target Preparation/Irradiation

Irradiation of a liquid target on a cyclotron includes the previous preparation of a target solution containing the enriched material, which benefits from the high yields provided by the same nuclear reaction employed in the case of a solid target. The target solution was obtained by the dissolution of nickel-64 metallic powder (95.0% enrichment) with 0.01 M nitric acid and was irradiated using an IBA Cyclone Kiube (IBA, Louvain-la-Neuve, Belgium) at the Institute for Nuclear Sciences Applied to Health (ICNAS Pharma), Coimbra, Portugal. The mass of enriched material dissolved in the irradiated liquid target varies between 50 and 100 mg, depending on the required activity. After irradiation, the solution is transferred to a post-processing hot cell under nitrogen pressure.

#### 3.3.2. Purification

Copper-64 automatic purification was conducted following the previously published methodology [15,16,29] using the Synthera^®^ Extension module (IBA, Louvain-la-Neuve, Belgium) and a dedicated disposable cassette, without any manual intervention. The purification process was completed within a maximum of 60 min from the EOB.

**Figure 6:**
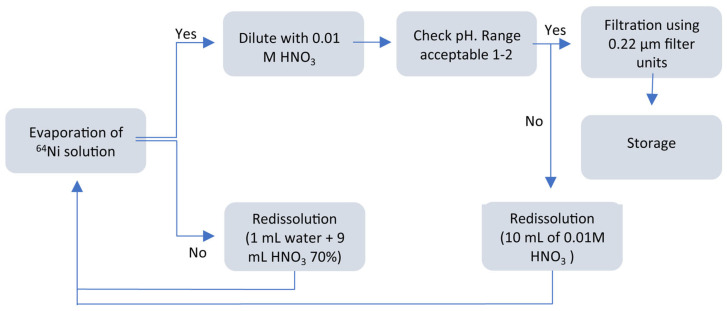
Recovery process for nickel-64 target material. Adapted from [23].

After purification, the recovered nickel-64 solution is reused. The nickel-64 target recovered from the CU resin waste container (~50 mL of diluted nitric acid solution) was evaporated and re-dissolved into the initial form of 10 mM HNO_3_. The recovery process is illustrated in Figure 6.

### 3.4. Preparation of Immunoconjugates

Trastuzumab (2 mg/mL) was incubated overnight with p-isothiocyanatobenzyl-diethylenetriaminepentaacetic (p-SCN-Bn-DOTA, 0.14 μmol) in 0.1 M sodium bicarbonate at pH 9 at 37 °C, using 20 times molar excess of DOTA. Conjugated DOTA-Trastuzumab was then purified by molecular exclusion on Sephadex G-25 column (PD-10) in order to remove the excess chelator and avoid interference with the radiolabeling process. The column was equilibrated with 20 mL of NH_4_OAc 0.25 M, the conjugation mixture was loaded into the column and, finally, the pure conjugated DOTA-Trastuzumab was collected in 3.0 mL of NH_4_OAc 0.25 M. The pure aliquot of 0.5 mL was collected and stored at −20 °C. A small fraction of DOTA-Trastuzumab pure was analyzed by SEC-HPLC to examine their structural integrity, using the same isocratic method described above. Nanobody ICNAS-01 was conjugated with two different chelating agents, NODAGA-maleimide and NOTA-maleimide. Before conjugation, a solution of Nanobody ICNAS-01 (1.05 mg/mL in PBS) was treated with dithiothreitol (DTT) in order to reduce spontaneously formed intermolecular disulfide bonds. In brief, 2 mL of stock Nanobody ICNAS-01 solution (1.05 mg/mL) was mixed with 30 μL of DTT solution (1 M) freshly prepared and incubated for 120 min at 37 °C. Thereafter, the mixture was applied to PD-10 size-exclusion column, pre-equilibrated, and eluted with NaCl 0.9%, as described above. The reduced Nanobody ICNAS-01 was submitted to an ultrafiltration concentrator tube Amicon^®^ Ultra (3 K) and then incubated with NODAGA- and NOTA-maleimide with a molar ratio of 1:10 (Nanobody:chelator) for both chelators for 90 min. Again, conjugated NODAGA-Nanobody and NOTA-Nanobody were purified as previously described, using NaCl 0.9% as eluent. The pure aliquots of 0.5 mL were collected and stored at −20 °C. A small fraction of both pure immunoconjugates was analyzed by SEC-HPLC to examine their structural integrity using the same isocratic method described above.

#### Determination of Protein Concentration of the Immunoconjugates

The protein concentration of the purified immunoconjugates–DOTA-Trastuzumab, NODAGA-Nanobody, and NOTA-Nanobody–were determined by the bicinchoninic acid method (BCA), using Albumin standard 2 mg/mL. In brief, copper (II) sulfate 4% (reagent A) was mixed with 50 volumes of bicinchoninic acid (BCA, reagent B). In a 96-well plate, 200 μL of the combined reagents (A and B) were added to three different immunoconjugates samples of 5 μL to be analyzed. For accurate determination of unknown protein concentration, a standard curve using different concentrations (2000, 1500, 1000, 750, 500, 250, 125, and 25 μg/mL) of bovine serum albumin (BSA) was prepared. The plate was incubated for 30 min at 37 °C to produce green-to-purple complexes. The absorbance was measured at 562 nm in ELISA equipment and the concentration of the protein was calculated.

### 3.5. Radiolabeling of Immunoconjugates

Varying quantities (38 to 280 μL) of [^64^Cu]CuCl_2_ (from liquid and solid target) were incubated with varying quantities of DOTA-Trastuzumab (285 or 780 μg), NODAGA-Nanobody (882 μg or 1.48 mg), and NOTA-Nanobody (497 μg or 1.22 mg) at room temperature (RT), pH 6 for 30 min.

After reaction, the mixtures were diluted to 1 mL with NaCl 0.9% and purified using a size exclusion PD-10 column pre-equilibrated with 20 mL of NaCl 0.9%. The mixture was loaded on the column, washed with 1.5 mL of NaCl 0.9%, and finally, each radiopharmaceutical, [^64^Cu]Cu-DOTA-Trastuzumab, [^64^Cu]Cu-NODAGA-Nanobody, and [^64^Cu]Cu-NOTA-Nanobody, was eluted with 3.5 mL of NaCl 0.9%. The radiolabeling yield of the reactions was assessed on iTLC-SG using 0.1 M sodium citrate as mobile phase and counted on a TLC plate reader. Final radiochemical purity and SA were determined using TLC and SEC-HPLC.

### 3.6. In Vitro Stability

The stability of ^64^Cu-conjugated molecules was evaluated under various conditions: in the presence of PBS, DTPA 50 mM, and in mouse serum, respectively. All stability measurements were quantified by iTLC, using 0.1 M sodium citrate as mobile phase.

The published protocol was followed with minor changes [29]. Briefly, 250 μL of the final purified solution containing each radiolabeled ^64^Cu-conjugated compound under study was added to 500 μL of each medium (PBS, DTPA 50 mM, or mice serum), and the mixtures were incubated at 37 °C. At different time points, aliquots were taken and measured using the iTLC methods. Regarding stability in mice serum, 60 μL aliquots were taken, and 180 μL of cold ethanol was added to precipitate the plasma proteins. The mixture was centrifuged at 4000 rpm and 4 °C for 10 min. The supernatant was separated and collected for iTLC analysis, and the activity was measured using a gamma well-counter. The incubation time for [^64^Cu]Cu-DOTA-Trastuzumab was 7 days, sampled at 1, 24, 48, 72, 96, 120, 144, and 168 h, while for the other two radiopharmaceuticals, [^64^Cu]Cu-NODAGA-Nanobody and [^64^Cu]Cu-NOTA-Nanobody, the incubation time was 12 h and the samples were collected at 1, 2, 4, 6, and 12 h.

## 4. Conclusions

Solid targets have been used for many years to produce medical radionuclides such as copper-64 using cyclotrons. A variety of starting materials and protocols for electroplating and dissolution of the irradiated targets are available and they can produce large quantities of high-quality radioisotopes. Nevertheless, the cost of installing and maintaining a solid target station prevents its widespread use in all cyclotron facilities.

Liquid targets, despite producing lower yields, present some practical benefits. The fully automated process eliminates the need for pre- and post- irradiation target preparation and simplifies the transfer of irradiated material to an automated chemistry module located inside a shielded hot cell, with an improvement in radioprotection conditions and a reduction in processing time and cost. Additionally, a high recycling efficiency can be obtained, with a minimal loss of nickel-64, even after several consecutive runs.

Regardless of the production route, the final [^64^Cu]CuCl_2_ solution is ready to be used for radiolabeling of monoclonal antibodies and nanobodies in compliance with current good manufacturing practice (cGMP). The radiolabeling of antibodies and nanobodies such as [^64^Cu]Cu-DOTA-Trastuzumab, [^64^Cu]Cu-NODAGA-Nanobody, and [^64^Cu]Cu-NOTA-Nanobody was successfully achieved in both solid and liquid targets cases, with high radiochemical purity and SA suitable for further in vivo pre-clinical imaging.

## Figures and Tables

**Table 1:** Summary of copper-64 production parameters for solid vs. liquid target irradiations.

Cyctroton	Solid Target	Liquid Target
	ACSI TR-19	IBA Cyclone^®^ Kiube
Nickel-64 (mg)	48 ± 1	100.6 ± 3.6
Integrated current [μA·h]	150 ± 0.2	230.7 ± 94.1
Number of runs	2	10
Proton energy [MeV] extracted/on target	14.2/11.7	18/16.9
Beam current [μA]	25 ± 1.2	54.5 ± 7.8
Beam time [h]	6	4.1 ± 1.3
Activity at EOB [GBq]	13.5 ± 0.5	2.8 ± 1.3

**Table 2:** Radiolabeling conditions used with [^64^Cu]CuCl_2_ solutions obtained either from solid or liquid targets. Radiochemical yield and SA determined by iTLC.

	Solid Target	Liquid Target
[^64^Cu]CuCl_2_ (μL)	38	90
[^64^Cu]CuCl_2_ (MBq)	94	108
**% Radiochemical purity**		
DOTA-Trastuzumab (μg)	285	281
NODAGA-Nanobody (μg)	882	881
NOTA-Nanobody (μg)	497	630
% Radiochemical yield (n.d.c.) *		
[^64^Cu]Cu-DOTA-Trastuzumab	100	88.5
[^64^Cu]Cu-NODAGA-Nanobody	100	93.57
[^64^Cu]Cu-NOTA-Nanobody	100	82.76
**% Radiochemical purity**		
[^64^Cu]CuCl_2_	100	100
**SA (MBq/μg)**		
[^64^Cu]Cu-DOTA-Trastuzumab	0.33	0.30
[^64^Cu]Cu-NODAGA-Nanobody	0.11	0.15
[^64^Cu]Cu-NOTA-Nanobody	0.19	0.12

* n.d.c.—non-decay corrected.

## Data Availability

Not applicable.

## Supplementary Materials

The following supporting information can be downloaded at https://www.mdpi.com/article/10.3390/molecules28124670/s1, Figure S1: iTLC of the [^64^Cu]CuCl_2_ solution after purification from (A) solid target and (B) liquid target. Figure S2: Radio-HPLC chromatograms of pure radioimmunoconjugates (A) ^64^Cu-DOTA-Trastuzumab, (B) ^64^Cu-NOTA-Nb, and (C) ^64^Cu-NODAGA-Nb. Table S1: Radiolabeling yields and radiochemical purity obtained from the solid and liquid target.
