# Supplementary material for: Antibody and Nanobody Radiolabeling with Copper-64: Solid vs. Liquid Target Approach
Source: Molecules. 2023 Jun 9;28(12):4670. doi: 10.3390/molecules28124670 (PMC10304849; doi:10.3390/molecules28124670)
Supplement: Supplementary file 1 [file molecules-28-04670-s001.zip › molecules-2413781-supplementary.pdf]

Supplementary Material

Antibody and nanobody radiolabeling with copper-64: solid *vs* liquid target approach

Ivanna Hrynchak [1], Diana Cocioabă [2,3], Alexandra I. Fonseca [1], Radu Leonte [2], Sérgio J. C. do Carmo [1,4,5], Roxana Cornoiu [2,6], Amílcar Falcão [1,7], Dana Niculae [2], and Antero J. Abrunhosa [1,4,5*]

[1] Institute for Nuclear Sciences Applied to Health (ICNAS Pharma), Polo das Ciências da Saúde, University of Coimbra, 3000-548 Coimbra, Portugal; ivanna.ua@icnas.uc.pt (I.H.); alexandrafonseca@icnas.uc.pt (A.I.F.); sergiocarmo@uc.pt (S.J.C.d.C.); amilcar.falcao@uc.pt (A.F.)

[2] Horia Hulubei National Institute for Physics and Nuclear Engineering (IFIN-HH), Radiopharmaceutical Research Centre, 077125 Măgurele, Romania; diana.cocioaba@nipne.ro (D.C.); radu.leonte@nipne.ro (R.L.); roxana.cornoiu@nipne.ro (R.C.); dana.niculae@nipne.ro (D.N.)

[3] Faculty of Physics, Doctoral School of Physics, University of Bucharest, 077125 Bucharest, Romania

[4] Coimbra Institute for Biomedical Imaging and Translational Research (CIBIT), University of Coimbra, 3000-548 Coimbra, Portugal

[5] Institute for Nuclear Sciences Applied to Health (ICNAS), University of Coimbra, 3000-548 Coimbra, Portugal

[6] Faculty of Chemical Engineering and Biotechnologies, Doctoral School of Applied Chemistry and Materials Science, University Politehnica of Bucharest, 011061 Bucharest, Romania

[7] Faculty of Pharmacy, University of Coimbra, 3000-548 Coimbra, Portugal

* Correspondence: antero@pet.uc.pt

A

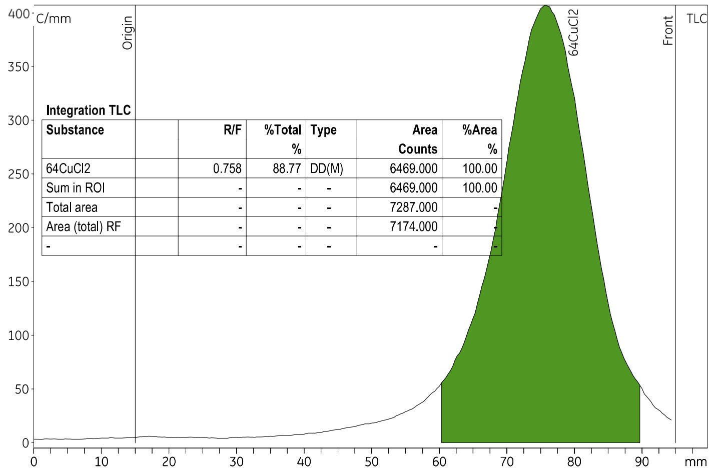

Integration TLC

Substance		R/F	%Total %	Type	Area Counts	%Area %
64CuCl2		0.758	88.77	DD(M)	6469.000	100.00
Sum in ROI		-	-	-	6469.000	100.00
Total area		-	-	-	7287.000	-
Area (total) RF		-	-	-	7174.000	-
-		-	-	-	-	-

B

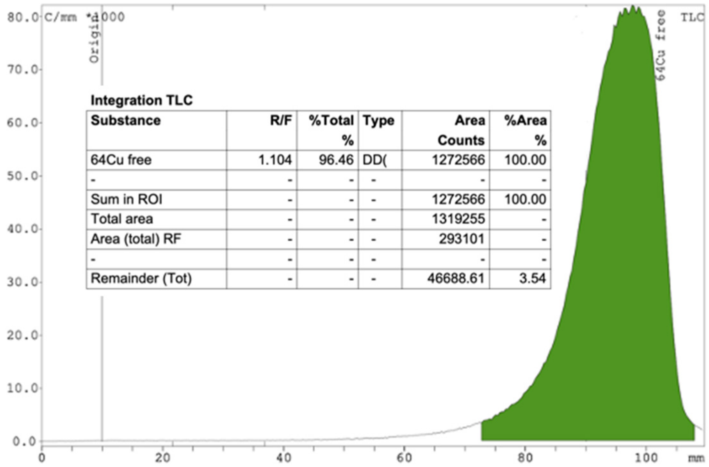

Integration TLC

Substance	R/F	%Total %	Type	Area Counts	%Area %
64Cu free	1.104	96.46	DD(	1272566	100.00
-	-	-	-	-	-
Sum in ROI	-	-	-	1272566	100.00
Total area	-	-	-	1319255	-
Area (total) RF	-	-	-	293101	-
-	-	-	-	-	-
Remainder (Tot)	-	-	-	46688.61	3.54

Figure S1. iTLC of the [^{64}Cu]$CuCl_2$ solution after purification from A) solid target and B) liquid target.

A

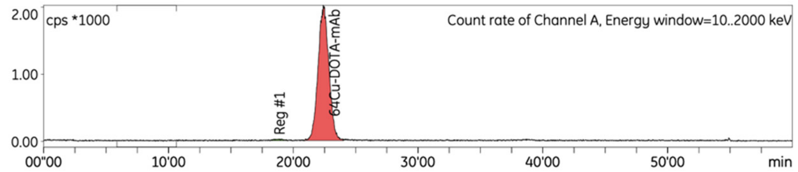

Integration Count rate of Channel A, Energy window=10..2000 keV

Substance	R/T s	Type	Area Counts	%Area %
Reg #1	18'53	DD(M)	1098.9	0.90
89Zr-DOTA-mAb	22'26	DD(M)	120407.1	99.10
-	-	-	-	-
Sum in ROI	-	-	121506.0	100.00
Area (total)	-	-	127935.5	-
BKG1 (CPS)	-	-	12.25	-
Remainder	-	-	6429.51	5.03

B

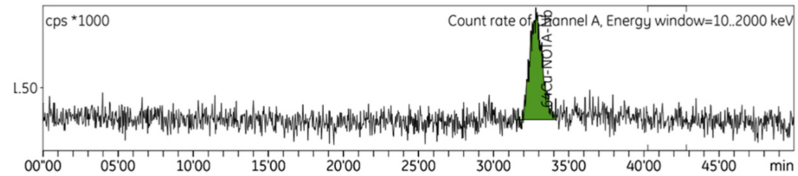

Integration Count rate of Channel A, Energy window=10..2000 keV

Substance	R/T s	Type	Area Counts	%Area %
64Cu-NOTA-Nb	32'52	DD(M)	30285.06	100.00
-	-	-	-	-
Sum in ROI	-	-	30285.06	100.00
Area (total)	-	-	28167.74	-
BKG1 (CPS)	-	-	1345.968	-
Remainder	-	-	-2117.32	-7.52

C

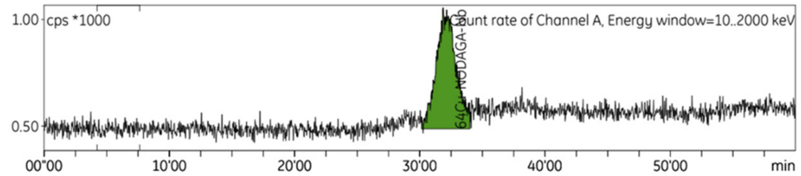

Integration Count rate of Channel A, Energy window=10..2000 keV

Substance	R/T s	Type	Area Counts	%Area %
64Cu-NODAGA-Nb	31'54	DD(M)	60945.54	100.00
-	-	-	-	-
Sum in ROI	-	-	60945.54	100.00
Area (total)	-	-	195617.76	-
BKG1 (CPS)	-	-	487.195	-
Remainder	-	-	134672.22	68.84

Figure S2. Radio-HPLC chromatograms of pure radioimmunoconjugates – A) [^{64}Cu]Cu-DOTA-Trastuzumab, B) [^{64}Cu]Cu-NOTA-Nb, and C) [^{64}Cu]Cu-NODAGA-Nb.

Table S1: Radiolabeling yields and radiochemical purity obtained from the solid and liquid target.

	Solid Target	Liquid target	
**Radiopharmaceutical**	**% Radiolabeling Yield by iTLC**	**% Radiolabeling Yield by iTLC**	**% Radiochemical purity after purification**
**[^{64}Cu]Cu-DOTA-Trastuzumab**	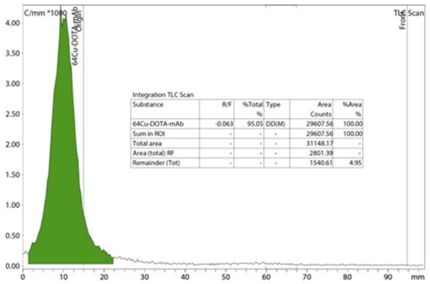	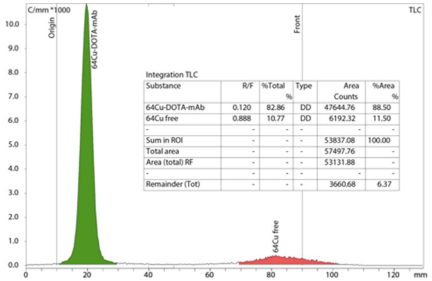	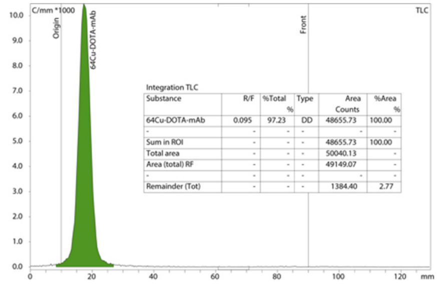
**[^{64}Cu]Cu-NODAGA-Nanobody**	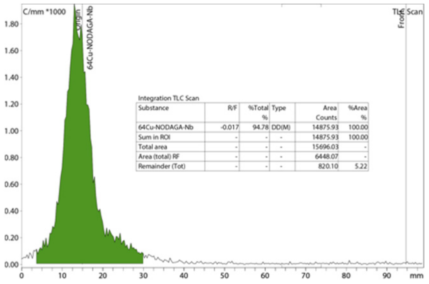	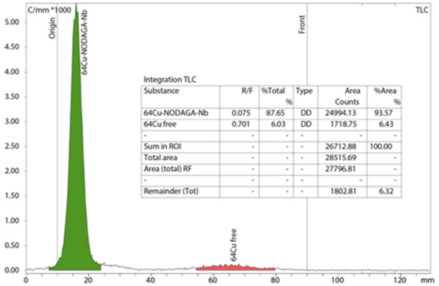	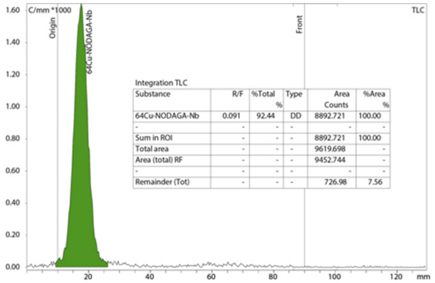
**[^{64}Cu]Cu-NOTA-Nanobody**	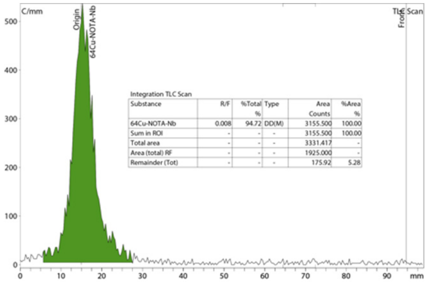	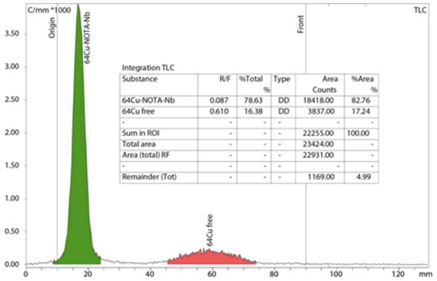	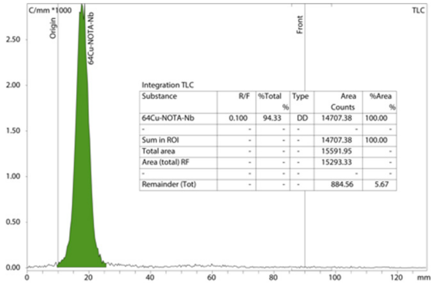
